# Eyespots in *Automeris* Moths Mimic Sympatric Owl Species (Lepidoptera: Saturniidae)

**DOI:** 10.1002/ece3.74144

**Published:** 2026-08-18

**Authors:** Chelsea Skojec, R. Keating Godfrey, Brandon Adams, Nabeeha Nazrul, Chandra Earl, Angela McTammany, Akito Y. Kawahara, Vaughn Shirey, Kate D. L. Umbers

**Affiliations:** ^1^ McGuire Center for Lepidoptera and Biodiversity, Florida Museum of Natural History University of Florida Gainesville Florida USA; ^2^ Department of Biology University of Florida Gainesville Florida USA; ^3^ Biology Department Middlebury College Middlebury Vermont USA; ^4^ Department of Physics University of Florida Gainesville Florida USA; ^5^ Department of Microbiology and Cell Sciences University of Florida Gainesville Florida USA; ^6^ National Ecological Observatory Network Biorepository Arizona State University Tempe Arizona USA; ^7^ Department of Interdisciplinary Studies University of Central Florida Orlando Florida USA; ^8^ Marine and Environmental Biology Section – Department of Biological Sciences University of Southern California California USA; ^9^ School of Science Western Sydney University Penrith New South Wales Australia

**Keywords:** artificial intelligence, machine learning, mimicry, predation

## Abstract

Eyespot mimicry is a well‐documented anti‐predatory strategy in many insects, particularly in moths. In the genus *Automeris*, species possess hindwing eyespots that resemble owl eyes, serving to startle the moth's predators. However, the mechanism by which *Automeris* eyespots function as a deterrent to moth predators is unresolved. Two hypotheses dominate the literature for eyespots broadly. First, that eyespots function as mimics of owl eyes with the rationale that the moth's predators are prey for owls and would avoid anything that looks like an owl. Alternatively, eyespots could be deterring due to their conspicuousness but are not mistaken for eyes. In this study, we investigate the mimicry hypothesis using species distribution models, artificial intelligence, and saliency mapping to assess whether *Automeris* eyespots are mimicking the eyes of sympatric owls. Geographic analyses reveal range overlap between *Automeris* species and co‐occurring owls, supporting the opportunity for mimicry‐driven selection. Our convolutional neural network (CNN) based confusability analysis demonstrates that *Automeris* eyespots are frequently misclassified as owl eyes, providing quantitative evidence for their visual similarity. Saliency heat map analyses on images of owls and moths showed high levels of confusion in the eyespot area and glimmer of moths and the eyes and beaks of owls. Our data suggest that *Automeris* eyespots are likely mimics, contributing to predator avoidance through deimatic behavior. This study highlights the potential for AI to provide new insights into the evolution of defensive traits in predator–prey interactions.

## Graphical abstract

Eyespot mimicry is a common anti‐predator strategy in moths, yet the mechanism underlying its effectiveness in Automeris remains unresolved. Using species distribution models, convolutional neural networks, and saliency mapping, we show that Automeris eyespots are visually confusable with sympatric owl eyes and that this similarity is concentrated in key visual features. This study highlights the potential for AI to provide new insights into the evolution of defensive traits in predator–prey interactions.

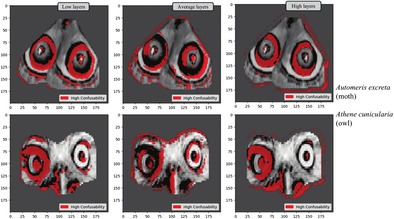

## 1 Background

Predator–prey interactions play a crucial role in the maintenance and diversity of defensive traits in a variety of animals. Insects are the primary food source for many vertebrates and have evolved a wide range of defenses to evade and protect against predation. This includes physical defenses such as urticating hairs, autotomy, or the production of noxious chemicals that make the insect unpalatable or dangerous to potential predators (Walker et al. 2023; Emberts et al. 2020; Kojima and Yamamoto 2020). These defenses may include aposematism, which requires previous encounters with the predator for learned avoidance to the defensive trait (Skelhorn, Halpin, and Rowe 2016; Skelhorn, Holmes, et al. 2016). In contrast, another common anti‐predatory defense in insects is camouflage or crypsis, the process of blending in with a surrounding environment to avoid detection by predators (Stevens and Ruxton 2019). A lesser‐known defense, deimatism, is a startle defense that combines both crypsis and aposematism (Drinkwater et al. 2022). Deimatic defenses are hidden at rest, but when prey is provoked, a display of color, sound, or movement is revealed. Deimatism is thought to help prey evade predation by confusing, disorienting, or startling a predator, giving prey time to retreat (Umbers et al. 2017; Olofsson et al. 2013). Whereas prey defenses such as aposematism rely on the learned avoidance over time from a potential predator, startle defenses exploit an innate fear response or reaction from a potential predator (Umbers et al. 2017). These defenses play a crucial role in the survival of insects and showcase the adaptations that have evolved in response to continuous pressure of predation.

**FIGURE 1:**
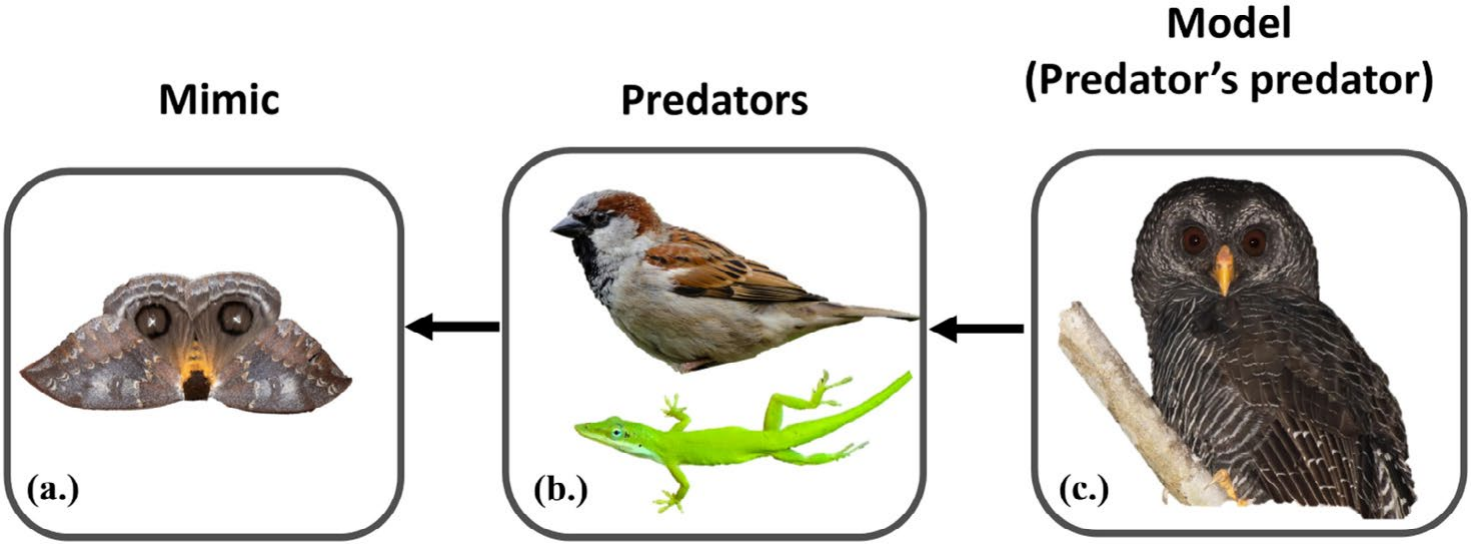
Conceptual mimicry model of *Automeris* and their predators. The hindwing eyespots of *Automeris* moths (a) resemble the eyes of owls (c). Owls are predators of a variety of small birds, rodents, and reptiles (b), which themselves predate on *Automeris*. *Automeris* moths mimic the eyes of their predators' predators, exploiting a fear response and reducing the likelihood of predation. Photo credits: Kristof Zyskowski (*Automeris denticulata*), Charles J. Sharp ( *Anolis porcatus* ), Mathias Appel ( *Passer domesticus* ), and Eduardo Borges ( *Strix huhula* ).

Eyespots are an elaborate adaptation found in many insects, and are thought to protect against predation by mimicking a potential predator's predator (Skelhorn, Halpin, and Rowe 2016; Skelhorn, Holmes, et al. 2016). Eyespots on the wings of some Lepidoptera may serve as a form of startle coloration or deimatic display, causing predators to be startled or intimidated by the sudden exposure of the eyespots (Blest 1957). In the genus *Automeris*, a majority of species have complex eyespot markings on their hindwings, with a strong resemblance to owl eyes. The hindwing eyespot's resemblance to owl eyes may also act as an additional component in a deimatic signal, mimicking the eyes of a potential predator and giving the impression that the prey is a threat (Figure 1; Vallin et al. 2011; Blut et al. 2012). This theory is supported by research conducted by Blest (Blest 1957), who found the closer the eyespots resemble actual eyes, the more protection they provide. However, there is differing research demonstrating the mechanism in which eyespots deter predators, and it remains largely unknown whether eyespots mimic the eyes of predators or act as conspicuous markings. Mimicry and conspicuous coloration may not be mutually exclusive, and Lepidopteran eyespots are known for serving multiple non‐exclusive functions such as deflection and sexual signaling (Kodandaramaiah 2009). It is possible that eyespots function as mimics in some species, while in others their primary role may be conspicuousness.

### 1.1 Eyespots as Predator Deterrents

Eyespots are comprised of concentric circles and vary in their complexity and apparent resemblance to the eyes of other animals, such as birds or larger predators (Stevens 2005). While some eyespots are less elaborate, containing one or two concentric circles such as the circles on the eyed click beetle ( *Alaus oculatus* ), other eyespots contain multiple defined circles with the visual complexity of an actual eye (Wong and Marek 2020). Research has focused on whether it is the conspicuous colors and shapes within the eyespots or the resemblance to an eye that makes eyespots an effective defense against predation (Stevens et al. 2009; De Bona et al. 2015; Olofsson et al. 2013). Several studies have found support for conspicuousness in eyespots to deter predation, not mimicry (Stevens et al. 2009, 2008). Stevens et al. (2007) found that artificial prey marked with black spots, white spots, or bars showed no differences in survival when contrast was controlled, and that more eye‐like patterns did not provide additional protective benefit. In their field experiments, artificial prey with different markings were placed on tree trunks and baited with mealworms; survival depended primarily on the contrast of the marking, not its resemblance to an eye. Eyespots can be conspicuous in shape, or contrast in color to make them stand out (Stevens et al. 2007; Mukherjee and Kodandaramaiah 2015). Additionally, circles have been found to be more effective at warding off predation than other salient shapes, such as triangles or squares (Stevens et al. 2008). Concentric circles with high internal pattern contrast were shown to be effective against predation without needing to resemble eyes (Stevens et al. 2008).

**FIGURE 2:**
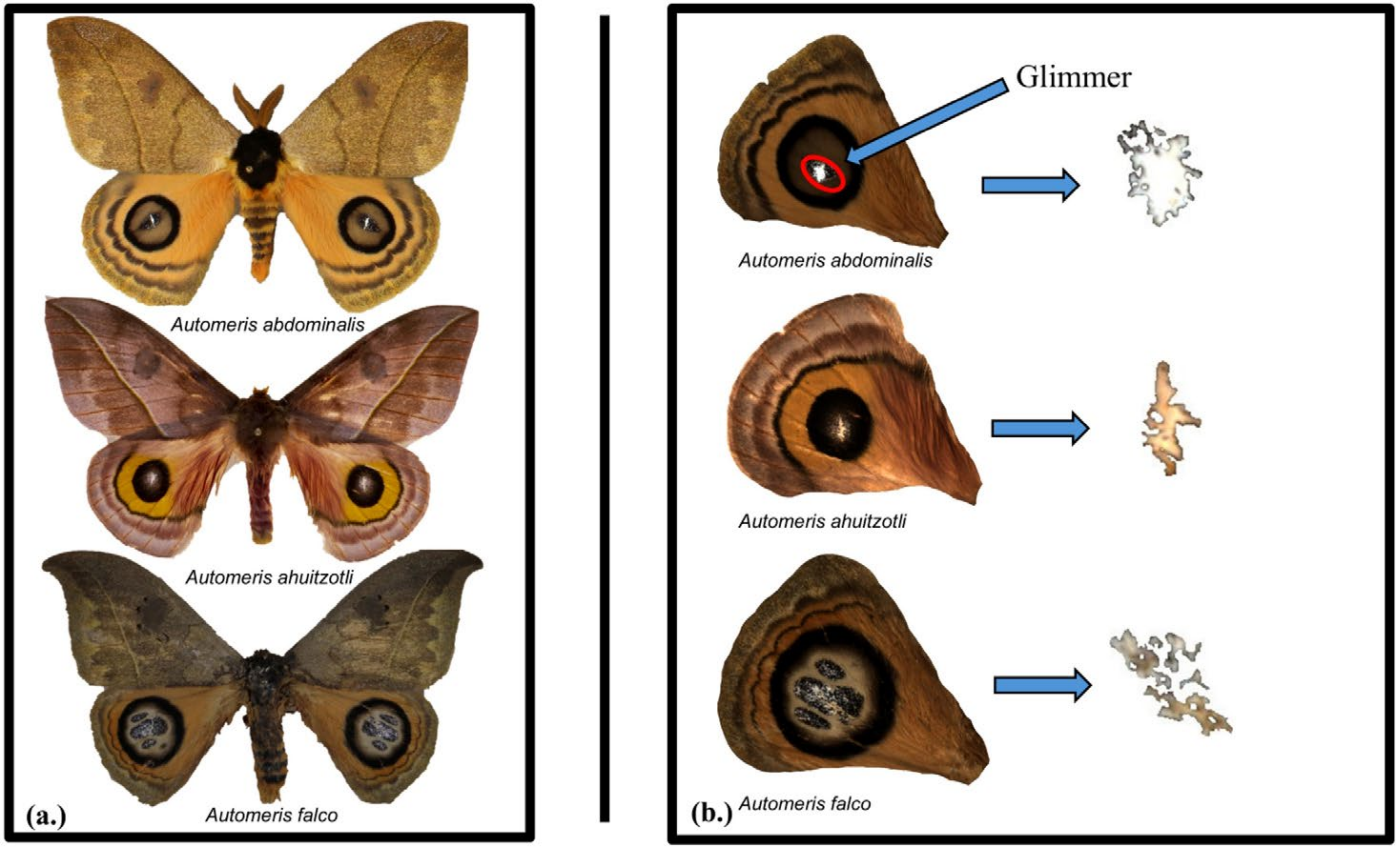
Glimmer variation in *Automeris* moths. (a) Full body images of *A. abdominalis* , *A. ahuitzotli*, and *A. falco* showing eyespots. (b) Hindwing and extracted glimmer regions highlight differences in size and shape of glimmer.

While some studies support the idea that eyespot defenses function primarily through conspicuousness, others provide evidence in favor of the mimicry hypothesis (Kodandaramaiah et al. 2009; Skelhorn and Rowland 2022). When testing for mimicry, previous research emphasizes the importance of testing eyespots with more elaborate and complex features to ensure the eyespots resemble an actual eye and not just a circle (De Bona et al. 2015). Such features include multiple concentric rings and the inner “glint” or “glimmer” within the center of an eyespot, thought to mimic the reflectance of light in a mammal or avian eye (Figure 2). In testing the efficacy of eye mimicry versus conspicuousness against avian predators, De Bona et al. (2015) found support for the eye mimicry hypothesis, not conspicuousness. In the study, when shown owl faces with eyes and owl faces without eyes, passerines had a higher aversion and startle to owl faces with eyes rather than without. When testing using elaborate features such as glimmer, behavioral studies testing the mimicry hypothesis found support for the mimicry hypothesis (Blut et al. 2012). Mimicry may also be more effective at deterring predators when eyespots are paired with background patterns that resemble the surrounding head area (Skelhorn et al. 2014). Inglis et al. (1983) found starlings had higher aversion when presented with a pair of eyes and an outlined head shape than just eyes alone. In the context of *Automeris*, the combination of the hindwing color, multiple concentric rings, and glimmer increases visual complexity by creating a 3‐dimensional looking eye, enhancing a resemblance to an owl's face, reinforcing the effectiveness of mimicry as opposed to conspicuousness. Although conspicuousness and mimicry are often treated as distinct hypotheses, they are not necessarily mutually exclusive, and given the diversity of predators and predation pressures faced by prey, it is likely that conspicuousness alone may be sufficient in some predator–prey interactions but not others. For the purpose of this study, we wanted to isolate and test the mimicry hypothesis. To do this, we developed a neural network classifier to test eyespot complexity and mimicry in *Automeris* moths, providing a potential new tool to test mimicry across ecological contexts.

Given that complex eyespots, and not singular circles, should be used in testing mimicry hypotheses, the genus *Automeris* is an ideal model system. Though species vary in eyespot complexity and elaboration, the majority display elaborate hindwing patterns of multiple concentric rings and glimmer. Many *Automeris* species occur in regions where their predators, such as small birds, rodents, and reptiles, are preyed on by owls (Marti 1974; Cadena‐Ortiz et al. 2022). Although eyespots may deter both vertebrate and insect predators (Prudic et al. 2015), the hypothesis that *Automeris* eyespots mimic owl eyes is most relevant to vertebrate predator interactions, which are the primary focus of this study. To the human eye, *Automeris* eyespots strongly resemble the eyes of owls. Studies have shown human vision can serve as a reliable initial proxy for observing patterns, color, and contrast, though it does not replicate avian tetrachromatic vision (Seddon et al. 2010; Bergeron and Fuller 2018). Therefore, our analyses should be interpreted as indirect measures of visual similarity rather than direct representations of predator sensory perception.

By mimicking owl eyes, *Automeris* moths may exploit innate fear responses in these predators and reduce their risk of attack. Recent phylogenetic analyses have clarified relationships within *Automeris* and provided a framework for studying defensive trait evolution in the genus (Skojec, Earl, et al. 2024). New genomic studies on *Automeris io* have laid the foundation for investigating genes involved in eyespots, wing patterning, and muscle movement used in deimatic displays (Skojec, Godfrey, and Kawahara 2024). Moreover, it is important to understand how these eyespots function within deimatic behavior to better evaluate their role in predator deterrence.

### 1.2 Deimatism and Eyespots

Deimatic behaviors include hidden and suddenly revealed displays that vary in form (Drinkwater et al. 2022), including bright red and blue contrasting colors under the tegmina of mountain katydids (Umbers et al. 2019), rhythmic motion accompanied by bright coloration and eye‐like spots under the wings of mantids (Vidal‐García et al. 2020), the yellow underbellies of many plethodontid salamanders via the unken reflex, and the red head flaps of the agamid lizard, *P. mystaceus* (Whiting et al. 2022). Though deimatic behaviors include a variety of colors, shapes, and movements that make up components of the defense, eyespots represent one possible visual component of these displays. In species with eyespots, these often only have one or two contrasting circles without elaborate features such as glimmer or multiple concentric rings, as seen in some mantids (Drinkwater et al. 2022). The elaborate eyespots found in *Automeris* suggest that eyespot complexity may contribute to the effectiveness of deimatic displays.

While there are many studies on eyespot mimicry, there is some conjecture around the mechanisms by which eyespots deter predators. It is likely some species have generalized eyespots that remain effective against predation without needing to truly mimic an eye of a predator. Conversely, some taxa may be true eyespot mimics, and that mechanism may be why their defense is effective (Vallin et al. 2011). Natural history, ecology, orientation of the eyespots, and the natural surroundings should all be considered in these investigations (Mizuno et al. 2024). Here we examine the resemblance between eyespots on *Automeris* moths throughout their geographic range and use convolutional neural networks (CNNs) to test visual similarity between owl eyes and eyespots using a standard confusability matrix to determine if some species mimic owls that live in the same regions or have generalized eyespots. Because convolutional neural networks analyze static images, our approach is not intended to distinguish behavioral components of deimatic displays. Rather, we use CNNs as a tool to quantify whether the visual features of *Automeris* eyespots resemble owl eyes more than expected under a hypothesis of conspicuous coloration alone. CNNs process images by detecting shapes, edges, and color contrasts, features which are relevant to early stage visual processing in biological systems (Cuthill et al. 2017). While CNNs do not replicate the cognition of a predator, their layered structure allows them to extract low and middle level features or patterns that are comparable to initial detection of visual signals. Initial detection is important in predatory prey interactions, as the decision to attack or retreat can be made within moments of detection (Stevens 2005).

If *Automeris* eyespots function as mimics of owl eyes, we expect to observe several patterns consistent with mimicry as the underlying protective mechanism. Complex eyespot features, such as multiple concentric rings and a central glimmer, have likely been maintained throughout the evolution of *Automeris* due to selective pressures from visually acute predators. Second, we anticipate species with more elaborate eyespots will exhibit greater geographic overlap with sympatric owl species, suggesting a potential ecological basis for mimicry. Finally, we predict that CNNs trained to classify visual features will frequently misclassify *Automeris* eyespots as owl eyes, particularly in species with significant geographic overlap. In contrast, CNN analyses of our control species lacking eyespots, *E. imperialis* , should have substantially lower confusability and higher accuracy scores in differentiating the two, lending support to the hypothesis that visual similarity to a predator model rather than general conspicuousness underlies the protective value of *Automeris* eyespots. To test whether the confusion arises from the eyespots and the owl eye regions, we expect saliency maps used to find which specific features on the owls and moths caused confusion to show high contrast in these regions.

## 2 Methods

### 2.1 Species Distribution Models

To determine the potential convergent evolution between specific *Automeris* and owl species, we determined species range overlaps among extant species.

To retrieve a list of owl species reporting in North and South America, we searched the Global Biodiversity Information Facility (GBIF; http://www.gbif.org) for all Strigidae and Tytonidae records occurring on both continents. The resulting species list was standardized to the BirdLife International (2022), in which both extinct species and incorrectly labeled non‐American species were removed. Bird range maps were requested from Birds of the World and then read into R. Any species with multiple range maps (often depicting seasonal or breeding ranges) were merged to create one range map per owl species.

*Automeris* species' range maps were created via species distribution modeling using records from both GBIF and the Symbiota Collections of Arthropods Network (SCAN). Although SCAN does contain records that might also be available on GBIF, many of the collections on SCAN are not made available. All *Automeris* records were retrieved from GBIF using the occ_search function from the rgbif v0.7.0 package (Chamberlain et al. 2022). *Automeris* occurrence records from SCAN were downloaded directly from https://scan‐bugs.org/, from all available collections. The resulting taxonomic list from the two datasets was standardized (Lemaire 2002), and some records not identified to species from the DNA and species database BOLD were also removed. The final dataset included 62 *Automeris* species and 76 owl species for downstream geographic overlap analyses. To increase the available records for spatial modeling, records without spatial information (i.e., without decimalLatitude and decimalLongitude fields) were considered for georeferencing. Species with low amounts of georeferenced occurrence records and available non‐georeferenced records were collaboratively georeferenced using the GEOLocate platform (https://www.geo‐locate.org/web/WebComGeoref.aspx). Remaining non‐georeferenced records were removed, and all records were merged and standardized to Darwin Core metadata format (Wieczorek et al. 2012).

Species distribution models for each species were built using a pipeline modified from Abbott et al. (2022). BioClim variables clipped to a shapefile of the Americas were used as potential predictors for the model (Booth et al. 2014). First, an accessible area (500 km) of potential species distribution was created by buffering the records of the species of interest. We chose a larger distance threshold for potentially accessible areas, given the scarcity of records for neotropical species. This allowed the restriction of species projections to areas surrounding recorded species records, resulting in more conservative species range maps. Occurrence records were thinned to remove duplicate records and to account for oversampling in predictor cells, thus leaving one record per grid cell. Predictors were clipped to the accessible area. Models were built using the ENMevaluate function from the ENMeval v2.0 package (Kass et al. 2021), designed to tune and evaluate models using Maxent (Phillips and Dudík 2008). The best performing model was reprojected back to the accessible area for the species of interest and converted to a binary classification using a threshold optimized for both the sensitivity and specificity of the species' model.

The percent overlap between *Automeris* range maps and owl range maps created from Bird of the World was calculated for all *Automeris*/owl species pairs. We then ordered pairs by percent overlap, inversely weighted by range size, so that smaller ranges carried more weight than larger ranges when overlapped. We did this to avoid overweighting widespread species, since large range owls and large range moths would naturally overlap with many species given the large area they cover. Inverse weighting allowed us to reduce this background overlap and identify more geographically specific sympatric species pairs, including finer scale regions where localized mimic model associations may occur.

### 2.2 Neural Networks

We trained several convolutional neural networks to process, validate, and analyze photographic data from *Automeris* spp. and owls taken on iNaturalist. We chose CNNs specifically as they are a staple for processing and analyzing images. We used iNaturalist photographs because they represent organisms in natural poses rather than artificial settings, such as in natural history museum collections. Images were selected from all available iNaturalist observations and were not filtered by time of day. Instead, images were subjected to a quality assurance pipeline, and only high‐quality images with front‐facing views and visible regions of interest were retained for analysis. Further, it was necessary to use iNaturalist images since owl eyes do not preserve well and rapidly lose color in collections or are replaced with glass eyes. Our data extraction and analysis pipeline consisted of a series of neural networks to (a) mask relevant regions of interest from both moth and owl photos, (b) assess the quality of those masks for downstream characterization, and (c) perform a confusability analysis using various parameterizations of convolutional neural networks to assess when moth‐owl confusability is elevated. We also utilized the open‐source Python package BioEncoder to represent each pair of moth wings and owl faces in a high‐dimensional embedding space (Lürig et al. 2024). We outline the procedure for each neural network below. We used neural networks as the basis of our analysis since they are adept at representing high‐dimensional features and complexity in images in a resulting numerical representation. Further, they allow us to rapidly process unstandardized images from iNaturalist and perform quality assurance checks on them. CNNs work by using a set of mathematical operators to extract “features” from images, learning those features and using them to either crop out target regions from images (regions of interest) or make classifications about what the image represents. Features are learned attributes or patterns in collections of pixels across the image; they are not predefined in our models.

The first task in our computer vision pipeline involved segmenting relevant regions of interest from iNaturalist photos of *Automeris* and owl species. We manually annotated 585 images of *Automeris* regions of interest (ROIs) and 598 images of owl face ROIs to perform this segmentation task. We used MakeSense.ai to construct these polygon annotations (Skalski 2025).

The second task, post‐segmentation, was a quality control and assurance check of the produced ROIs from the iNaturalist images. Image quality criteria were defined a priori, including visibility of both owl eyes, intact forward‐facing wings of *Automeris*, and sufficient resolution to resolve eyespot features. From these ROIs, high‐quality and low‐quality images were manually sorted by humans into different folders based on these criteria. The folders were then used to train the neural net on high and low‐quality images of *Automeris* segments and images of owl eye segments. Images were sorted into these folders for training and validation. For owls, training used 131 high‐quality images and 374 low‐quality images. For *Automeris*, training used 203 high‐quality images and 739 low‐quality images. High‐quality images of *Automeris* were forward‐facing wings with intact eyespots, and high‐quality owls were forward‐facing with both eyes visible and high resolution. For validation *Automeris*, 198 high‐quality images and 772 low‐quality images were used. Owls validation used 77 high‐quality images and 320 low‐quality images. Once the neural network was able to differentiate high vs. low‐quality images, we were able to parse 4081 owl images and 1233 *Automeris* images to be used later in the confusability and geographic analysis.

Third, using the images that passed this quality control filter, we programmed a confusability‐test framework with various neural networks to determine when confusability between *Automeris* and owls was highest. We define confusability as the proportion of images misclassified between categories, meaning *Automeris* images classified as owls and owl images classified as *Automeris*. To do this, we parameterized six neural network infrastructures: (a) low features (4 convolutional filters) and no hidden layers (1 output layer); (b) low features and one hidden layer (with 4 nodes); (c) average features (16 convolutional filters) and no hidden layers; (d) average features and one hidden layer; (e) high features (32 convolutional filters) with no hidden layers; and (f) high features with one hidden layer. Multiple layers were used to increase the complexity of patterns that the CNN can record. The more layers used in a CNN, the more complex features it interprets; this was to ensure the complexity of eyespot shapes were accounted for. Additionally, hidden layers were used to make predictions from the patterns from convolutional layers. We trained each of these neural networks 10 times, using 1914 images for training and 1030 images for validation.

Following the training of the moth‐owl classifier, we deployed a binomial GLM (logit‐link) to assess whether or not the (a) number of convolutional layers, (b) number of neural layers, and (c) the inclusion of Imperial moths ( *Eacles imperialis* ), a control group lacking eyespots, were significant in predicting the model's confusability in misclassifying moths as owls. The model was specified using the form defined in Equation (1), below (note the use of interaction effects throughout):

(1) $$\begin{array}{c} p\left(\text{moth classified}\;\mathrm{as}\;\mathrm{owl}\right)\sim \text{Convolutional Layers}\\ \times \text{Neural Layers}\times \text{Inclusion of Imperial Moths} \end{array}$$

Finally, to examine if localized adaptation may play a role in *Automeris* wing similarity to owl faces, we transformed the *Automeris* and owl image data into a representative embedding space using BioEncoder (Lürig et al. 2024). An Imperial moth ( *Eacles imperialis* ) control group without eyespots was used to test the accuracy of neural net confusability in owls and *Automeris* moths. Unlike *Automeris*, *E. imperialis* lacks eyespots, providing a baseline comparison to test confusability. Following the same methods for annotation and training as *Automeris* wings, 699 *E. imperialis* hindwings were annotated and split into training and validation groups before being run through the neural network.

Our CNN architectures varied in feature complexity in order to capture different levels of visual detail. This allowed us to test *Automeris* eyespots and owl eye confusability under multiple processing conditions.

To further uncover whether or not the patterning on *Automeris* wings matched with our hypotheses that eyespot complexity and glimmer mimic features in owl eyes, we produced heatmaps for highly confusable features based on our CNN training. To do this, we calculated Shannon entropy from the predicted output ($\text{Entropy}=-\left(p\times \log\left(p\right)+\left(1-p\right)\times \log\left(1-p\right)\right)$, where p, is the value of the sigmoid classifier, when p=0, the classifier is completely confident that the resulting image is a moth, when p=1 it is confident it is an owl) (Shannon 1948). Shannon entropy was maximized when the model was most uncertain of placing the image in one class or the other (i.e., classifying the image as either a moth or an owl). Using this we then sliced through the last convolutional layer to identify features that contributed to predictive uncertainty. For visualization, we truncated these features to the top 90% of features most strongly contributing to confusion. These heatmaps allowed us to qualitatively assess where the most confusability was happening in the resulting classifier.

To ensure the CNN confusability between owl eyes and *Automeris* eyespots were from those specific features in the images it analyzed, saliency heat mapping was also conducted. Saliency maps were generated using two CAM‐produced heat maps that highlight the features in the photos that contributed most to classification uncertainty between moth and owl images.

## 3 Results

**FIGURE 3:**
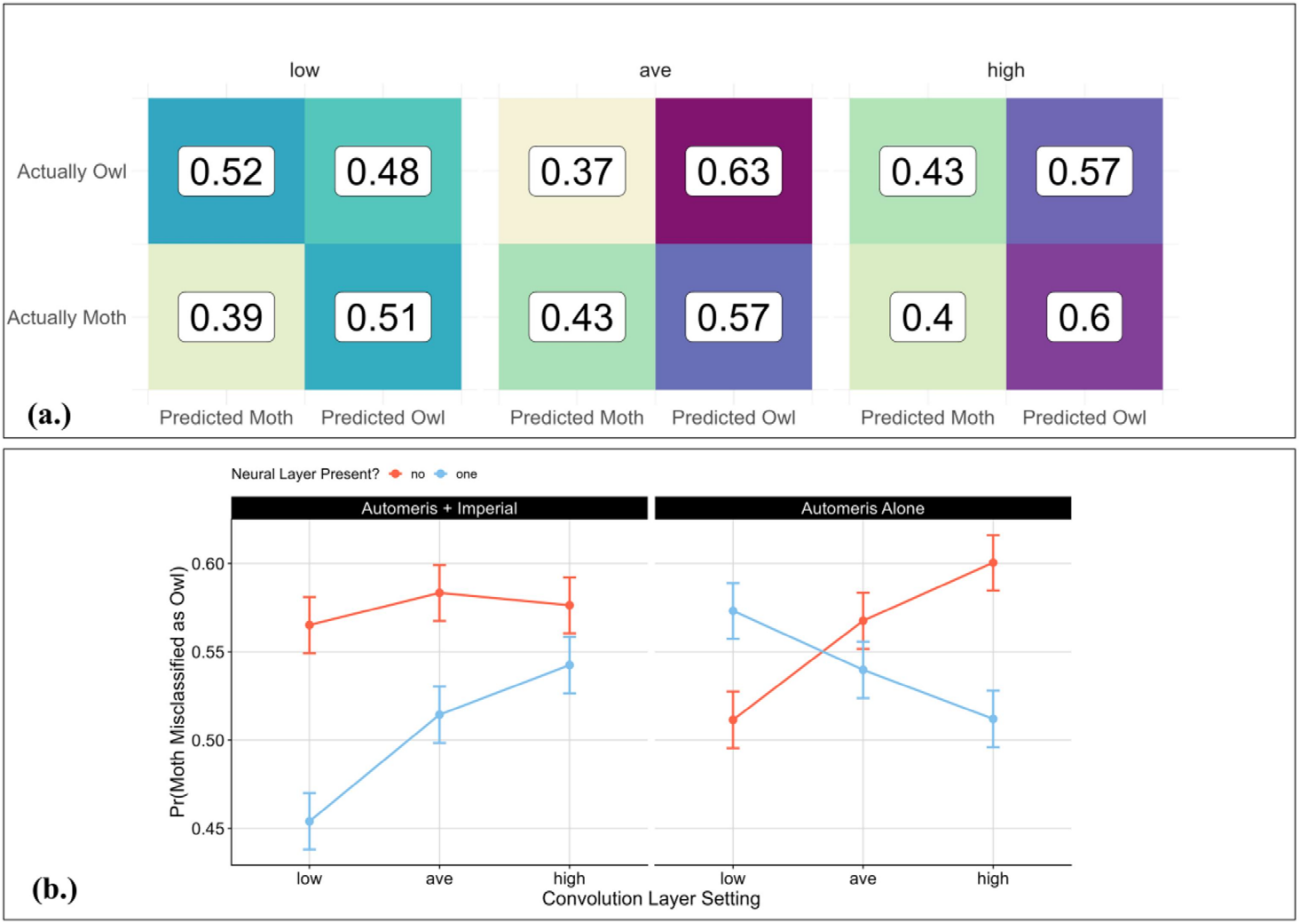
Neural network confusability and validation accuracy for distinguishing *Automeris* moth eyespots from owl eyes. (a) confusion matrices showing prediction outcomes for three neural networks with low, average, and high numbers of convolutional filters. Higher filter counts did not reduce confusability, with owl eyes frequently misclassified as moth eyespots and vice versa. (b) Results from a generalized linear model that contained the three‐way interaction between the number of convolutional layers, the number of neural layers, and the inclusion of the Imperial moth training data in predicting the probability that a moth image would be misclassified as an owl.

**FIGURE 4:**
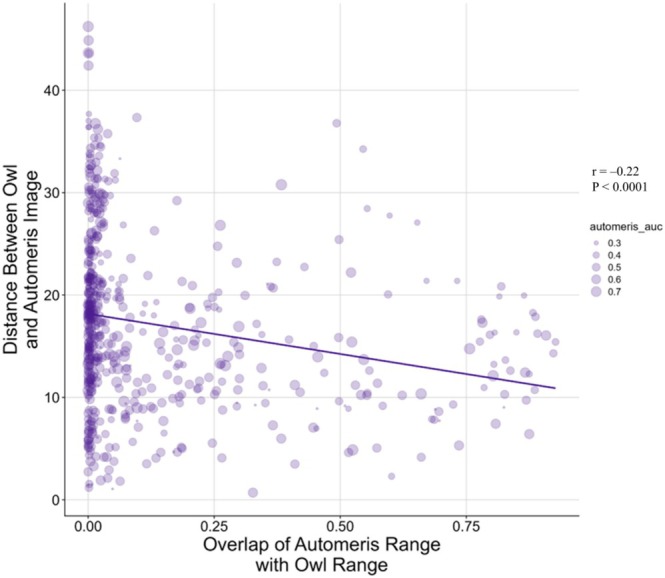
Correlation of distance between owl and *Automeris* images in the BioEncoder embedding space and the overlap in range between *Automeris* and owl species. Distance in the embedding space becomes smaller (more similar images) when overlap between ranges is greater. Points are weighted by *Automeris* SDM AUC. The correlation coefficient was *r* = −0.22, *p* < 0.0001.

**FIGURE 5:**
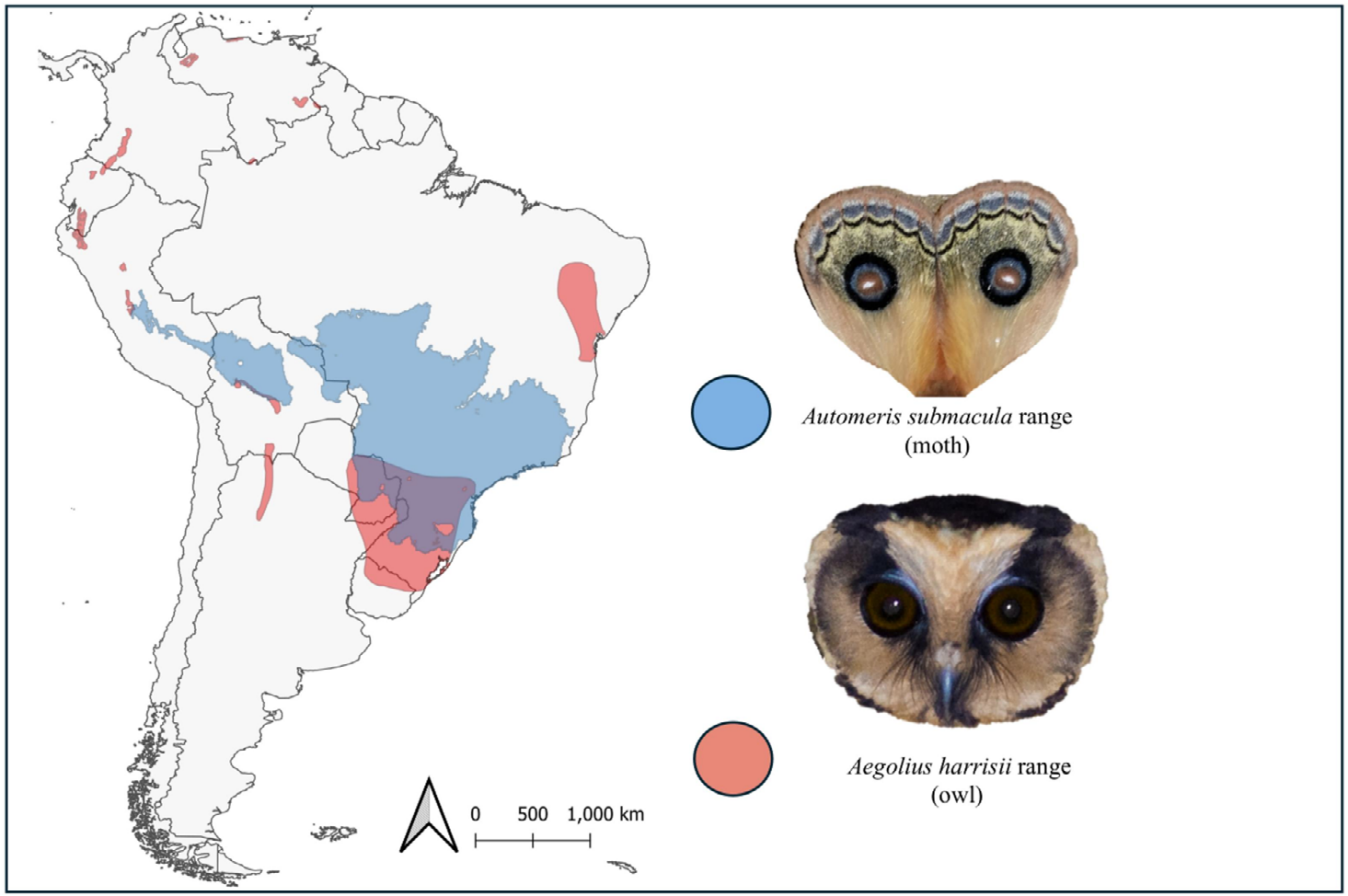
Example best match owl and *Automeris* species from pairwise comparisons of image features and geographic range. The blue area represents the range of *Automeris submacula* (moth), and the red areas are the distribution of *Aegolius harrisii* (owl). Owl photo credit: Romilson Lopes, moth photo credit: Ronald Péret (CC BY).

Our confusability analysis for the highest complexity models indicated 60% of the *Automeris* moth images were categorized as owls, with a mean confusability of 58%. This was in line with our prediction that *Automeris* eyespots would be frequently misclassified as owl eyes. For *E. imperialis* versus owls, the median accuracy score was 87.5%, demonstrating that moth wings without eyespots have a higher level of accuracy than wings with eyespots. The results of our GLM are consistent with our ecological expectations of how the study system operates (Table 1). In a scenario where the model distinguishing owls from moths is trained using *Automeris* alone, increasing the complexity of patterning leads the model, with no neural layers, to become increasingly confused. In contrast, a model with one neural layer becomes better able to distinguish between moths and owls (Figure 3b, Table 1). The inclusion of Imperial moths in the training dataset imparts a different effect on confusability. Model performance with no neural layer remains consistent while the inclusion of a neural layer increases confusability among models that learn more complex patterns. Boxplots of model validation accuracy show that confusion increased for *Automeris* even with increasing model complexity, while the control species *E. imperialis* , which lacks eyespots, remained consistently distinguishable (Table S1, Figure S1). By combining species distribution model overlap with image feature similarity, we were able to compare geographic co‐occurrence with visual similarity (mimicry). As geographic range overlap increases, *Automeris* species exhibit greater visual similarity to their corresponding owl species. Pairs with no range overlap were excluded, which strengthened the correlation. Pairwise comparisons of the distance of image features embedding similarity in high‐dimensional space and overlap in range between owls and *Automeris* moths revealed significant patterns (Figures 4 and S2). This allowed us to identify the best‐matching owl and *Automeris* species pairs (Figure 5). The correlation was weighted by the *Automeris* SDM AUC (area under the curve) metric, with a correlation coefficient of −0.22, a T statistic of −5.34, and a *p*‐value less than 0.0001. As range overlap increases, distances in the embedding space decrease, indicating greater visual similarity between images.

**TABLE 1:** GLM coefficients (binomial GLM). Results indicate that the model significantly predicts the probability of confusion for moth images incorrectly classified as owls. A visual interpretation of the model is included in Figure 3b.

	Estimate	Std. error	z value	*p*
Intercept	0.26246	0.03299	7.957	< 0.001
ConvLayers (avg)	0.07438	0.04678	1.59	0.112
ConvLayers (high)	0.04584	0.04672	0.981	0.327
NeuralLayers (one)	−0.44694	0.04655	−9.602	< 0.001
*Automeris* alone (TRUE)	−0.21646	0.04646	−4.66	< 0.001
Convolutional layers (avg) × Neural layers (one)	0.16787	0.06586	2.549	0.011
Convolutional layers (high) × Neural layers (one)	0.3091	0.06587	4.692	< 0.001
Convolutional layers (avg) × *Automeris* alone	0.15188	0.06594	2.303	0.021
Convolutional layers (high) × *Automeris* alone	0.31585	0.06609	4.779	< 0.001
Neural layers × *Automeris* alone	0.69611	0.0658	10.579	< 0.001
Convolutional layers (avg) × Neural layers × *Automeris* alone	−0.5296	0.09309	−5.689	< 0.001
Convolutional layers (high) × Neural layers × *Automeris* alone	−0.91783	0.0932	−9.848	< 0.001

**FIGURE 6:**
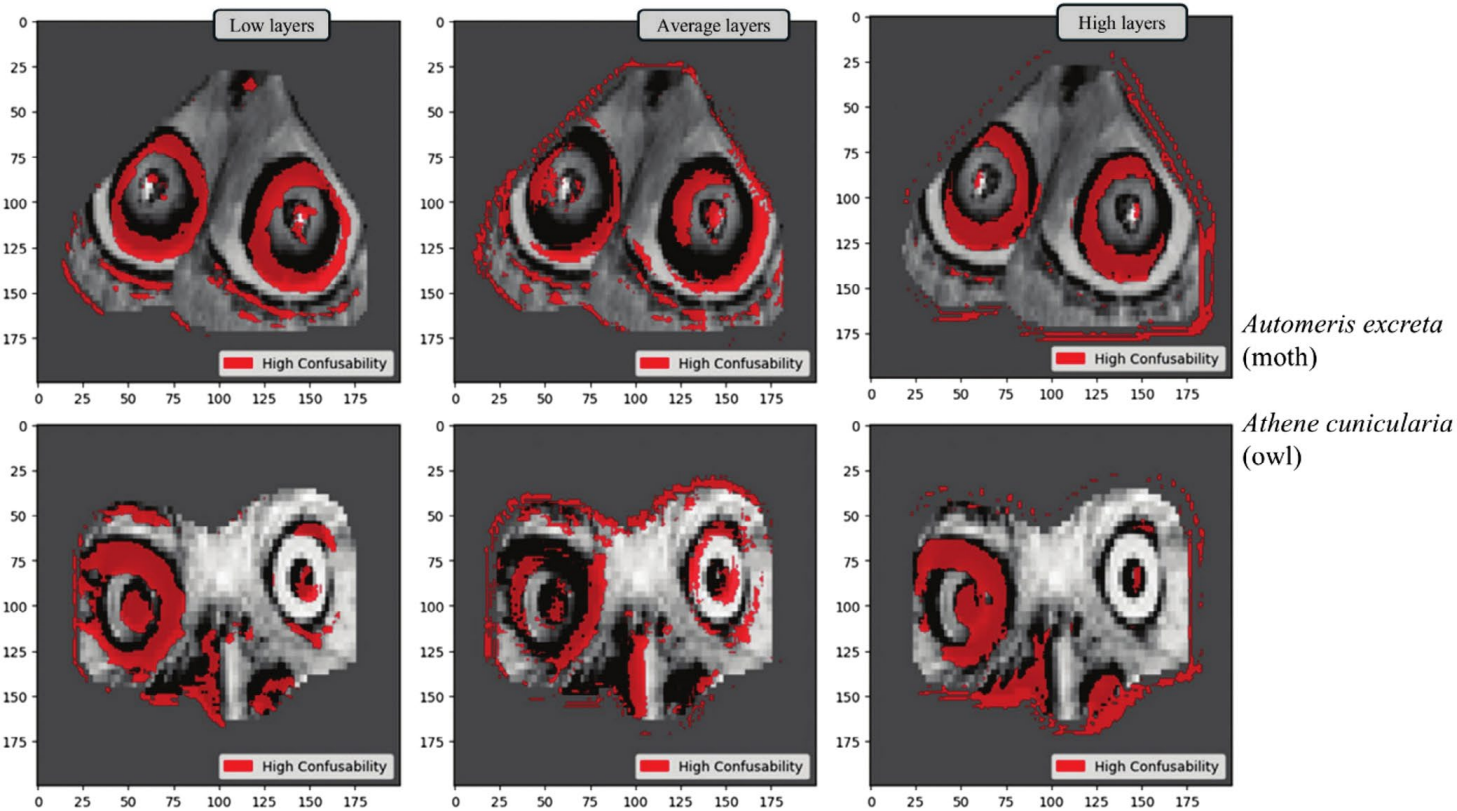
Saliency heat map showing regions with the highest confusability/entropy for the convolutional neural networks with low, average, and high numbers of convolutional layers in *Automeris excreta* (moth) and *Athene cunicularia* (owl) species.

To further investigate which image features contributed most strongly to owl and *Automeris* confusability, saliency heat maps were generated from the CNN models (Figure 6). For *Automeris*, the heatmap highlighted multiple concentric rings and the inner glimmer. The results closely mirror one another, with the outer rings around the owl eye matching the outer ring of the eyespots, and the owl eye matching the center eyespot and glimmer. These results suggest that CNN confusability is driven by these corresponding regions.

## 4 Discussion

These findings reinforce that *Automeris* eyespots are likely mimics rather than solely conspicuous markings. The complex features of eyespots, including concentric rings and the central glimmer, appear sufficient to trigger misidentification in neural networks. The confusion matrices were generated from networks trained with varying convolutional filters; even as model complexity increased, the networks continued to confuse *Automeris* eyespots with owl eyes (Figure 3). This confusability is consistent with elaborate patterning in *Automeris* functioning as a mimicry signal rather than simply a conspicuous deterrent. Our results do not exclude a role for conspicuousness, but instead demonstrate that mimicry provides a strong additional explanatory framework for these patterns.

The association between geographic overlap and increased visual similarity observed in *Automeris* is consistent with patterns reported in other mimicry systems. In *Heliconius* butterflies, local mimicry rings and regional convergence are maintained by frequency dependent selection and geographic structure (Mallet and Gilbert Jr. 1995; Merrill et al. 2015). Similar patterns have been described in Papilio swallowtails, where mimicry evolves in response to geographically variable model communities (Kunte 2009; Puissant et al. 2023). Viceroy butterflies also show geographic variation in mimicry. Across their range, viceroy wing coloration and mimicry dynamics vary in association with local *Danaus* species, including monarchs and queens (Ritland 1998; Prudic et al. 2019). Similar geographic variation has also been reported in poison dart frogs, where populations resemble different model species in different regions (Twomey et al. 2014). These examples suggest that mimicry can vary across a species range and may be influenced by the distribution of locally occurring models. Our results indicate that *Automeris* eyespots may follow a similar pattern, with increased sympatry with owl species associated with greater visual similarity.

Although *Automeris* eyespots strongly resemble owl eyes to humans, other visually acute predators like diurnal snakes and raptors may also increase selective pressure on eyespot morphology. Predation on moths occurs under both diurnal and nocturnal conditions (Ruxton et al. 2019). We focus on owls because our hypothesis is whether *Automeris* specifically mimics owls, they are widespread, and found across the range of *Automeris* with many species overlap. Additionally, owls prey on small birds and reptiles that themselves predate on *Automeris*, creating potential selection pressure from multiple types of predators at the same time (Ratcliffe and Nydam 2008; Janzen et al. 2010). Further, we specifically chose owls as they have forward facing eyes with concentric rings that are visually a close match to *Automeris* eyespots, whereas snakes and other reptiles lack forward‐facing eyes and have less concentric rings in and around their eyes.

Recreating the natural environment and dynamic of predator–prey encounters using both real live predators and live prey individuals is often difficult and sometimes improbable given the location and availability of live animals. In studies that manage to acquire both, there are often other biases and ecological factors to consider (van Wilgenburg and Elgar 2013). Additionally, it is important when testing traits predicted to be used as a defense to present them in their natural state. For example, *Caligo* butterfly eyespots are often compared to owl eyes, though in their natural state they rest with closed wings, showing only one side of the butterfly and therefore only one eyespot (Mizuno et al. 2024). Given these challenges, researchers have increasingly turned to artificial or simulated approaches to anti‐predatory behaviors. Previous studies have used computer modified experiments to recreate real life behavioral scenarios, including a computer game using human subjects to pick which square objects were more startling (Loeffler‐Henry et al. 2018). In another study, human participants as visual foragers were asked to relocate and click animated digital prey moving on a computer to study the effects of flash display on visual continuity disruption (Bae et al. 2019).

For this study we did not believe artificial intelligence would be directly comparable to thought or decision making in predators, but that artificial intelligence provides insight specifically into initial predator feature detection of complex shapes and colors. Given that convolutional neural networks rely on a number of layers that focus on specific attributes, these layers can be adjusted to capture different levels of feature complexity relevant to initial visual detection. This study illustrates how AI‐based neural networks can be used to investigate initial detection of visual signals. The way in which shapes, colors, contrast, and complexity are initially perceived between similar objects is likely an area in which AI can provide significant insight in the future. Although AI may not be comparable to avian or reptilian predator intelligence, future behavioral studies should explore different ways neural networks can be used to study perception in sensory systems. As additional observations are added to iNaturalist, *Automeris* species will become better represented both taxonomically and geographically. Future studies will be able to include a broader range of *Automeris*, with more precise models of species pairs that could not be included in this study due to limited observational data.

## 5 Conclusion

This study provides support for eyespot mimicry as a primary anti‐predatory mechanism in *Automeris* moths. By using evolutionary, ecological, and AI‐based analyses, our findings highlight how elaborate defenses may evolve to exploit predator detection of visual signals, reinforcing mimicry as a strategy in predator–prey dynamics and the potential creative uses of AI to explore and answer long time biological questions.

## Author Contributions

**Chelsea Skojec:** conceptualization (lead), data curation (lead), formal analysis (lead), investigation (lead), methodology (lead), project administration (equal), validation (lead), visualization (lead), writing – original draft (lead), writing – review and editing (lead). **R. Keating Godfrey:** conceptualization (supporting), formal analysis (supporting), investigation (supporting), methodology (supporting), validation (supporting), visualization (supporting), writing – original draft (supporting), writing – review and editing (equal). **Brandon Adams:** data curation (supporting), investigation (supporting), writing – review and editing (supporting). **Nabeeha Nazrul:** data curation (supporting), methodology (supporting), writing – review and editing (supporting). **Chandra Earl:** data curation (supporting), methodology (supporting), writing – original draft (supporting). **Angela McTammany:** data curation (supporting), methodology (supporting), writing – original draft (supporting). **Akito Y. Kawahara:** investigation (supporting), project administration (supporting), supervision (equal), writing – review and editing (supporting). **Vaughn Shirey:** conceptualization (supporting), data curation (equal), formal analysis (equal), investigation (supporting), methodology (supporting), software (equal), supervision (equal), validation (equal), visualization (equal), writing – original draft (equal), writing – review and editing (equal). **Kate D. L. Umbers:** conceptualization (equal), investigation (supporting), project administration (supporting), supervision (equal), writing – original draft (supporting), writing – review and editing (supporting).

## Funding

This material is based upon work supported by the National Science Foundation. Graduate Research Fellowship under Grant No. 00130513 to C.S.

## Conflicts of Interest

The authors declare no conflicts of interest.

## Supporting information


**Figure S1:** Boxplots of validation accuracy across model complexity levels for *Automeris* (blue) and control *Eacles imperialis* (yellow). *E. imperialis* showed higher accuracy, while *Automeris* accuracy declined with increasing complexity, reflecting visual similarity between moth eyespots and owl eyes.
**Figure S2:** Example worst match owl and Automeris species from pairwise comparisons of image features and geographic range. The black area represents the range of Bubo magellanicus (owl), and the yellow areas are the distribution of Automeris janus (moth). Owl photo credit: Julia Ariadna (CC BY), moth photo credit: Angie Nobre de Moura.
**Table S1:** Analysis of Deviance (binomial GLM).

## Data Availability

All data supporting the findings of this study are available in the Dryad Digital Repository at: https://doi.org/10.5061/dryad.gmsbcc315.
