# Supplementary material for: Eyespots in Automeris Moths Mimic Sympatric Owl Species (Lepidoptera: Saturniidae)
Source: Ecol Evol. 2026 Aug 18;16(8):e74144. doi: 10.1002/ece3.74144 (PMC13482772; doi:10.1002/ece3.74144)
Supplement: Supplementary file 1 [file ECE3-16-e74144-s001.docx]

Figure S1. Boxplots of validation accuracy across model complexity levels for *Automeris* (blue) and control *Eacles* *imperialis* (yellow). *E. imperialis* showed higher accuracy, while *Automeris* accuracy declined with increasing complexity, reflecting visual similarity between moth eyespots and owl eyes.

Figure S2. Example worst match owl and *Automeris* species from pairwise comparisons of image features and geographic range. The black area represents the range of *Bubo magellanicus* (owl), and the yellow areas are the distribution of *Automeris janus* (moth). Owl photo credit: Julia Ariadna (CC BY), moth photo credit: Angie Nobre de Moura.

|  | **Df** | **Deviance** | **Residual Df** | **Residual Deviance** | **P-value** |
| --- | --- | --- | --- | --- | --- |
|  |  |  | 119 | 5416.6 |  |
| Convolutional Layers | 2 | 34.188 | 117 | 5382.4 | < 0.001 |
| Neural Layers | 1 | 90.859 | 116 | 5291.5 | < 0.001 |
| *Automeris* Alone | 1 | 5.952 | 115 | 5285.6 | = 0.015 |
| Convolutional Layers × Neural Layers | 2 | 10.633 | 113 | 5274.9 | = 0.005 |
| Convolutional Layers × *Automeris* Alone | 2 | 10.661 | 111 | 5264.3 | = 0.005 |
| Neural Layers × *Automeris* Alone | 1 | 32.072 | 110 | 5232.2 | < 0.001 |
| Convolutional Layers × Neural Layers × *Automeris* Alone | 2 | 97.954 | 108 | 5134.2 | < 0.001 |

**Table S1.** Analysis of Deviance (binomial GLM).
